# Casting light on the national mission to eliminate sickle cell disease in India

**DOI:** 10.1002/hem3.70033

**Published:** 2024-10-19

**Authors:** Frédéric B. Piel, Roshan Colah, Dipty L. Jain

**Affiliations:** ^1^ Department of Epidemiology & Biostatistics School of Public Health, Imperial College London London UK; ^2^ National Institute of Immunohaematology Indian Council of Medical Research Mumbai India; ^3^ Department of Paediatrics Shalinitai Medical College and Research Institute Nagpur India

Sickle cell disease (SCD) is a neglected global public health burden.^1^ Although it primarily affects populations from sub‐Saharan Africa,^2^ SCD is also prevalent across the Indian subcontinent, particularly among tribal (or scheduled) populations.^3^ India is the most populated country in the world. According to the latest population estimates of the United Nations World Population Prospects,^4^ its population includes 1.441 billion people, and it is expected to further increase to reach 1.697 billion in 2063. India ranks as the country with the third highest number of annual births affected by SCD, after Nigeria and the Democratic Republic of the Congo.^2^ Although SCD has long been considered to be mild across the Indian subcontinent, recent evidence has demonstrated that there was a much wider range of severity than previously thought.^5^ Finally, tribal populations tend to be largely over‐represented in the low socio‐economic groups across India, making them a vulnerable group for many communicable and non‐communicable diseases.^6^

Interventions to reduce SCD morbidity and mortality, such as newborn screening, vaccinations, penicillin prophylaxis, and hydroxyurea, have proven to be effective in large‐scale studies in high‐ and upper‐middle‐income countries, including the United States,^7^ United Kingdom,^8^ Jamaica,^9^ and Brazil.^10^ Pilot studies of these interventions have been conducted in numerous low‐income countries.^11^ Cost‐benefit analyses conducted in sub‐Saharan Africa^12^ and India^13^ suggested that these interventions would also be effective in these settings. Nevertheless, due to a lack of political and financial commitments, no national program has so far been launched in a low‐ or lower‐middle‐income country of high prevalence for SCD. Despite the curative promises of gene therapies,^14^ there is an urgent need to scale up interventions in the most affected countries to improve the quality of life of patients affected and reduce the global burden of SCD.^11^

In July 2023, the Government of India launched the “National Sickle Cell Anaemia Elimination Mission.”^15^ Although this program was officially launched by Prime Minister Modi, it did not receive much attention internationally. The stated aims of the Mission are twofold: (i) to improve the care of all SCD patients for their better future and (ii) to lower the prevalence of the disease by 2047 through a multifaceted coordinated approach toward screening and awareness strategies. The ambitious plan at launch was to screen 70 million people across India over the first 3 years of the Mission. The screening was at first intended to target primarily 0–18‐year‐olds, before incrementally including individuals up to 40 years of age.

Screening methods recommended for the Mission are either the solubility sickling test, with positive cases to be confirmed by gold‐standard high‐performance liquid chromatography (HPLC), or rapid point‐of‐care testing (POCT) devices which have emerged in the last decade. The solubility test does not allow for differentiation between homozygotes (SS), with SCD, and heterozygotes (AS), with sickle cell trait (SCT). Moreover, it suffers from a high rate of false negatives when utilized for newborn screening due to the presence of a high amount of fetal hemoglobin (HbF) and when HbS represents less than 10% of the total hemoglobin. As a result, in high‐income countries, the solubility test tends to be used only for emergency screening. HPLC validation tends to be expensive and relies on highly skilled staff, sophisticated equipment requiring regular maintenance, and available reagents. It is therefore possible that confirmatory tests are not systematically conducted to validate results from the solubility sickling test. POCT devices potentially offer a promising alternative, particularly outside large cities. Best practices currently recommend positive POCT tests to be validated by a laboratory method, such as HPLC or electrophoresis.^16^ Current POCTs can usually differentiate between SCD and SCT, but they cannot identify sickle cell—β thalassemia. Recent developments of the Gazelle^TM^ device aim to address this limitation.^17^ The sensitivity and specificity of some POCT devices, such as SickleScan^TM^ or HemoTypeSC^TM^, have been validated in rigorous national^18^, ^19^ and international studies.^20^ To be self‐reliant and reduce costs, devices used for the Mission are primarily manufactured in India. Although several screening methods have been officially validated by the Indian Council of Medical Research (ICMR), the sample sizes were relatively small and several tests have a claimed sensitivity and specificity of 100%. There is an urgent need for independent validation of these methods, and of POCT devices, in particular, using internationally recognized standard protocols.

The Mission has driven substantial national initiatives to produce POCT devices locally, at much lower costs than products currently on the market anywhere in the world. Given that people identified with SCD need to be managed, a similar impact has been observed for the local production of hydroxyurea, the main drug currently used to prevent complications in SCD. If confirmed reliable and potent, these efforts could have major implications for the future of screening and managing SCD globally and across sub‐Saharan Africa in particular.

Daily screening data from the Mission are freely available in an online dashboard.^21^ As of September 17, 2024, a total of 42,139,843 individuals have been screened across India, with 163,765 identified as SCD (0.39%) and 1,144,274 as SCT (2.72%) (Table 1). Confirmation of the screening results was shown as still under process for another 398,628 tests (0.95%). The dashboard does not provide a regional or ethnic breakdown, but these preliminary data provide valuable insights into the scale of the Mission in India.^22^ Just over 1 year after its launch, the Mission has already screened more than 42 million people. For comparison, this is more than 10 times the annual number of babies tested as part of the universal newborn screening program in the United States (~3.6 million) and 70 times that number in the United Kingdom (~600,000). The scale of this initiative means that, on average, almost four hundred new cases of SCD are potentially identified every day by the Mission. This is roughly equivalent to the number of new births affected identified over a year in the United Kingdom. In addition, about 2500 carriers are also identified daily. Although these individuals are not of concern from a clinical perspective, they are highly relevant to the future burden of SCD and should be informed about the disease and offered premarital screening^23^ and genetic counseling.^24^

**Table 1:** Screening data from the dashboard of the national mission to eliminate sickle cell disease in India (https://sickle.nhm.gov.in/home/guest_dashboard). Accessed on September 17, 2024.

	HbAA	HbAS	HbSS	Unconfirmed	Total
Observed	40,433,175	1,144,274	163,765	398,628	42,139,843
%	95.95%	2.72%	0.39%	0.95%	100%
Expected^a^	40,282,384	1,445,856	12,974	/	41,741,214
%	96.51%	3.46%	0.03%	/	100%

a Based on the Hardy–Weinberg Equilibrium, not taking the “Unconfirmed” into consideration.

Data from the Mission suggest an allele frequency of 1.76%, which is slightly lower than previous national‐level estimates.^22^ Using this frequency and the generic assumptions of the Hardy–Weinberg Equilibrium (HWE),^25^ the expected number of individuals with SCD (12,974) would be almost 12‐fold smaller than the one observed (163,765). Surveys of adults in sub‐Saharan Africa tend to find much fewer people with SCD than expected due to the high infant mortality associated with SCD.^26^ Although the difference between the observed and the expected seen in India is likely to be due to a complex range of factors, there is a high level of consanguinity in India and in tribal populations, in particular.^27^ The dashboard data suggest an inbreeding coefficient (*F*)—a standard measure of consanguinity in clinical genetics—of 0.021, which is consistent with previous studies.^28^ Although preliminary data from the Mission suggest that preventing consanguineous marriages alone could potentially result in dramatic reductions in the annual number of SCD births across India. This would also impact the prevalence of various other health outcomes including congenital anomalies. This observation raises important ethical issues around the role of genetic counseling and prenatal diagnosis in India.^29^ Furthermore, it is essential to understand cultural practices and traditions across such a large and diverse country, particularly when considering tribal populations which are often marginalized and over‐represented in the lower socio‐economic status groups. Evidence from the Pakistani communities in the United Kingdom suggests that changes tend to occur slowly, over generations, even in multi‐ethnic communities outside their country of origin.^30^ Similarly, the compulsory programs implemented in Saudi Arabia highlight the complexity of such interventions in a country with a much smaller population and substantial financial resources.^31^

Screening is a necessary first step to identify patients with SCD and to manage them and their complications. It is nevertheless essential that the education material, healthcare facilities, specialist staff, and treatment options are available and accessible to all to successfully follow up with individuals identified with SCD and ideally carriers too. Several guidelines and training materials have been produced in Hindi and English as part of the Mission,^32^ but little information is currently available on their use. No data is publicly available yet on the impact of the Mission, but it will be key to its success to track follow‐up rate, uptake of hydroxyurea, and changes in the frequency of complications and the perception of the disease as a result of this national initiative. Reliable data will be essential to demonstrate the benefits and ensure the sustainability of the Mission. Only 5% of the total population of India would have been screened after the first 3 years of the Mission. There are about 25 million births per year across India, so it would require a sustained effort over the entire country to achieve a universal newborn screening program equivalent to those currently in place in the United States or the United Kingdom. If this is the long‐term objective, this will require sustained political will and financial resources.

The vocabulary used in such a large‐scale initiative is important and needs to be carefully considered. The term “elimination” used for this Mission is reminiscent of ambitious strategies used for infectious diseases, including malaria.^33^ Considering the genetic nature of SCD, this terminology is unfortunate. The Mission should be used as an opportunity to reduce inequalities affecting the populations of India and to reduce the future burden of SCD across the subcontinent, rather than to further stigmatize tribal populations or to potentially feed eugenics propaganda.

In conclusion, our aim here is to highlight both the scale of this initiative and the need for reliable data to monitor its acceptability and impact. It is remarkable that a program of such a scale could be implemented so quickly across a country so large and populated as India. More than 40 million individuals screened in just over 1 year for a largely neglected disease is a considerable achievement and this should be celebrated. Nevertheless, rigorous processes need to be in place to ensure that screening devices and protocols meet international standards and that guidelines are consistently followed across the country so that high‐quality epidemiological data are generated. The national and international credibility of the Mission relies on reliable data. Cost‐benefit analyses will also need to be conducted to demonstrate the impact of this initiative. Finally, the success of this Mission could have significant implications to demonstrate the feasibility of implementing large‐scale programs on SCD in sub‐Saharan African countries.

## AUTHOR CONTRIBUTIONS

Frédéric B. Piel wrote the first draft of the manuscript. Frédéric B. Piel collected data and performed statistical analysis. Frédéric B. Piel, Roshan Colah, and Dipty L. Jain all reviewed the manuscript.

## CONFLICT OF INTEREST STATEMENT

The authors declare no conflict of interest.

## FUNDING

Frédéric B. Piel acknowledges support from an Imperial‐India Connect Fund award.

## Data Availability

Data used were freely available from the Mission's dashboard: https://sickle.nhm.gov.in/home/guest_dashboard.
